# Instigating prevalent abiotic stress resilience in crop by exogenous application of phytohormones and nutrient

**DOI:** 10.3389/fpls.2023.1104874

**Published:** 2023-02-09

**Authors:** Rinny Swain, Smrutishree Sahoo, Mamata Behera, Gyana Ranjan Rout

**Affiliations:** ^1^ Department of Agricultural Biotechnology, Crop Improvement Division, School of Agriculture, Gandhi University of Engineering and Technology (GIET) University, Rayagada, Odisha, India; ^2^ Department of Genetics and Plant Breeding, Crop Improvement Division, School of Agriculture, GIET University, Rayagada, Odisha, India; ^3^ Department of Agricultural Biotechnology, College of Agriculture, Odisha University of Agriculture and Technology, Bhubaneswar, Odisha, India

**Keywords:** abiotic stress, phytohormone, nutrient, signaling, antioxidant, gene expression

## Abstract

In recent times, the demand for food and feed for the ever-increasing population has achieved unparalleled importance, which cannot afford crop yield loss. Now-a-days, the unpleasant situation of abiotic stress triggers crop improvement by affecting the different metabolic pathways of yield and quality advances worldwide. Abiotic stress like drought, salinity, cold, heat, flood, etc. in plants diverts the energy required for growth to prevent the plant from shock and maintain regular homeostasis. Hence, the plant yield is drastically reduced as the energy is utilized for overcoming the stress in plants. The application of phytohormones like the classical auxins, cytokinins, ethylene, and gibberellins, as well as more recent members including brassinosteroids, jasmonic acids, etc., along with both macro and micronutrients, have enhanced significant attention in creating key benefits such as reduction of ionic toxicity, improving oxidative stress, maintaining water-related balance, and gaseous exchange modification during abiotic stress conditions. Majority of phytohormones maintain homeostasis inside the cell by detoxifying the ROS and enhancing the antioxidant enzyme activities which can enhance tolerance in plants. At the molecular level, phytohormones activate stress signaling pathways or genes regulated by abscisic acid (ABA), salicylic acid (SA), Jasmonic acid (JA), and ethylene. The various stresses primarily cause nutrient deficiency and reduce the nutrient uptake of plants. The application of plant nutrients like N, K, Ca, and Mg are also involved in ROS scavenging activities through elevating antioxidants properties and finally decreasing cell membrane leakage and increasing the photosynthetic ability by resynthesizing the chlorophyll pigment. This present review highlighted the alteration of metabolic activities caused by abiotic stress in various crops, the changes of vital functions through the application of exogenous phytohormones and nutrition, as well as their interaction.

## 1 Introduction

Feeding the global population rise which is soon to reach 2.3 billion by 2050 is a challenging task in every way, so a considerable increase in grain productivity to at least about 70% is the need to accomplish this global challenge efficiently (Tilman et al., 2011). However, the major drawback in achieving this objective is the frequent occurrence of abiotic stress which affects the plant’s metabolic activities and triggers the biosynthetic pathways ultimately reflected in the reduction in quality and yield loss. Plants show their own mechanism to overcome the period of abiotic stress, for which maximum of their energy synthesized by the plant becomes diverted towards creating resistance or tolerance to the stress condition. The abiotic stress includes drought, cold, salinity, heat, water logging, metallic stress, etc. in plants transferring the energy to prevent the plant from such stresses and maintain normal growth. In the current scenario, these abiotic stressors are the major factors affecting production and productivity. Amongst various abiotic stresses, high temperature, water scarcity, and salinity are the most widespread and significant ones (Wani et al., 2013).

Plant body is a complex of several biomolecules, among them phytohormones are the molecules produced in very low concentrations, however, they show their active participation in regulatory activities (Shabir et al., 2016). The cellular activities are mostly regulated by the chemical communication inside the plant body with low-volume phytohormones (Vob et al., 2014). Phytohormones are most important to regulate various signal transduction pathways during abiotic-stress response. They regulate external as well as internal stimuli (Kazan, 2015). Auxin, cytokinin (CK), gibberellic acid (GA), ethylene, abscisic acid, brassinosteroids, salicylic acid, jasmonates, and strigolactones are the major phytohormones that have the major network in plant growth and development as well as in alleviating abiotic stress in plants. Nutrients are another crucial component that can minimize the effect of abiotic stress in plants by maintaining the inner homeostasis of the cell. Plant nutrients are considered the available form of food for plants for their normal growth and development. The plant nutrients are grouped into primary nutrients like nitrogen (N), phosphorus (P), and potash (K); secondary nutrients like calcium (Ca), magnesium (Mg)and sulfur (S); micronutrients like boron(B), zinc (Zn), iron (Fe)conditions, copper (Cu); and other beneficial nutrients like cobalt (Co), selenium (Se), silicon (Si). Due to global climate change, plant suffers a lot from nutrient deficiency. It was also noted that nutrient deficiencies are the major cause of yield loss during abiotic stress. Hence, proper nutrient management can elevate abiotic stress conditions in plants to some extent. Plant nutrients can mitigate stress also by activating stress resistance genes, enhancing antioxidant enzyme activity, creating osmoprotectant in cells, synthesizing heat shock proteins and other proteins related to stress tolerance, decreasing ROS activities, creating membrane stability, repairing DNA, enhancing chlorophyll content in leaves, reducing the uptake of heavy metals in the plant.

## 2 Effects of abiotic stress on plants

Abiotic stresses cause disorders in plants like osmotic stress in cells, retardation in cell development, reduced photosynthetic activity, seed dormancy, and late reproduction, and eventually show a negative effect on yield (**Table 1**). Among different types of abiotic stresses, water-deficit stress is most frequent in nature and causes ample of damage to crop plants. The rigorous impact of water deficit stress is due to reduced plant relative water content which causes osmotic and oxidative stress (Diouf et al., 2018). This condition occurs in salinity stress also and further triggers the same effect as drought (Munns and Tester, 2008). Both the drought and salinity stress the most menacing global abiotic stresses, which force a series of morphological, physiological, and molecular changes in plants, and in order to survive they require osmotic adjustment, ROS detoxification stomata closure, and cellular signaling (Diouf et al., 2018). Among the other stressors, high temperature can impact plants’ hormone production, nutrient uptake, stomatal conductance, transpiration rate, photosynthetic activities, enzymatic activity, antioxidants level, membrane stability index and reactive oxygen species (ROS) production (Hussain et al., 2018). Similarly, chilling stress in plants can also affect by putting impacts on tissue water content, membrane fluidity, and chlorophyll content (Zhang et al., 2012).

**Table 1:** Common responses of plants under abiotic stress conditions.

Types of stress	Effect on plant	References
Drought	Increases in leaf yellowing and senescence, leaf drooping, wilting, scorching of leaves, leaf rolling and brittleness, closed flowers and flower sagging, leaf etiolation, and premature fall of leaves.	Ruehr et al., 2019
Salinity	Ion toxicity, osmotic stress, nutrient deficiency, oxidative stress on plants, leaf area and chlorophyll content reduction, altered stomatal conductance, limited water uptake, and cell death,	Shrivastava and Kumar, 2015
Water logging	Inhibition of root respiration, blocked gas exchange between soil and atmosphere, accumulation of toxic substances, leaf stomata closure, chlorophyll degradation, leaf senescence, and yellowing, the decline in photosynthetic rate, inhibition of germination, nutrient deficiencies, inadequate ATP production, ROS production, chlorosis and necrosis in waxy leaves and yield reduction	Jiawei et al., 2021
Chilling/frost injury/cold stress	Reduced water potential, ice crystal formation leads cell and plant death, membrane destabilization, altered membrane permeability, destruction or degradation of chlorophyll, photosynthetic inhibition, cell expansion inhibition, cell death, tissue browning, blackening, wilting or curling of leaves and stems, disruption of conversion of starch to sugar, decrease CO_2_ exchange, disturbed mating system and yield reduction.	Mayland and Cary, 1970; Salvi et al., 2021
High temperature/heat stress	Inhibition of seed germination, increased oxidative stress, water loss, alteration in phenology, improper growth and development, alteration in photosynthesis, pollen grain sterility, improper seed setting, reduced shoot, and root growth scorching of leaves, branches and stems leaf senescence and abscission, fruit discoloration, and altered dry matter accumulation, reduced yield in plants	Hasanuzzaman et al., 2013

### 2.1 Drought stress

About half of the global arid and semi-arid regions are affected by drought stress. Under the conditions of drought stress, photosynthesis, growth, and physio-biochemical processes of plants are highly disrupted, which inhibits plant growth and development and results in yield loss. A significant loss in total biomass and productivity has resulted due to water stress conditions. Many researchers have reported that oxidative stress from excessive ROS i.e. superoxide, hydroxyl ions, nitric oxide, singlet oxygen production, and nutrition imbalance, altered cell membrane balance and biomolecules like DNA, proteins, and lipids, imbalanced photosynthetic efficiency reduced turgor pressure, and alterations in leaf gas exchange rates as some of the harsh impacts due to drought (Perveen and Hussain, 2020; Sofy et al., 2021; Alam et al., 2021; Zandi and Schnug, 2022). Numbers of morphological characteristics of plants, including seed germination, plant height, relative root length, root diameter, the total biomass of leaves and roots, number of leaves/plants, number of branches/plants, etc. are negatively impacted by drought stress (**Table 2**) which are more or less observed in every crops. Among the physiological impacts, crop plants experience partial stomatal closure and an increase in photorespiration due to an imbalance in carbon metabolism during water stress (Hu et al., 2019). Additionally, during stress, plants produce more reactive oxygen species (ROS), which harms chloroplasts through oxidation. All of these factors work together to limit photosynthates, which eventually lowers agricultural productivity. In response to the deadly impacts of water stress, plants activate their natural defense systems including various morphological, physiological, and biochemical adaptations, leaf rolling, altered leaf angle, deep root system, drought-resistant epigenetic phenotypic plasticity and gene activation, production of osmolytes, soluble proteins, proline, soluble sugars, and glycine betaine, etc. (Ozturk et al., 2021; Ghafar et al., 2021). While considering the effect of drought on phytohormones, the impact of stress depends on balancing of IAA and ABA content (Krishnan and Merewitz, 2014). Rapid ABA accumulation has also been observed under salinity and heat stress (Xiong et al., 2001). Experimental evidence regarding the exposure of moderate drought on *Triticum aestivum* and *T. spelta* showed initial increased accumulation of ABA and SA, decreased level of GA_3_ and IAA, alteration of CKs in roots and shoots (Kosakivska et al., 2022). ABA and ethylene significantly reduced gas exchange parameters, chlorophyll a and b content in cotton (Pandey et al., 2003).

**Table 2:** Impacts of drought stress on some major crops.

Crop	Effect	Reference
Wheat	Spikelet fertility and grain filling reduced crop yields and quality	Grzesiak et al., 2019
	Reduced leaf area	Naz and Perveen, 2021
Rice	Poor seedling germination	Liang et al., 2021
	Reduced leaf area	Naz and Perveen, 2021
Pea	Poor seedling germination	Al-Quraan et al., 2021
	Reduces nitrogen fixation	Gonzalez et al., 2001
Maize	Seedling germination	
	Reduced number of leaves	Ahmad et al., 2019
	Reduced hypocotyl length and fresh and dry weight of roots	Hu and Chen, 2020
	Decreased seed oil content	Ali et al., 2010
*Phaseolus vulgaris*	Drop in the dry weight of the shoot	Widuri et al., 2018
Soybean	Reduces nitrogen fixation	Serraj, 2003
	Decreased oil content up to 12.4%, reduction in oleic acid content	Dornbos and Mullen, 1992
Common bean	Altered Fe, Zn, P, and N nutrient concentrations, decreased in total protein content	Ghanbari et al., 2013
Chickpea	Altered ABA levels and seed-filling rate	Sehgal et al., 2018
*Nicotiana tabacum*	Chlorophyll pigments affected	Hu et al., 2018

### 2.2 Salinity stress

Saline soil having a high concentration of soluble salts with an ECe value of 4 dS/mL or higher in the soil. Salinity in the soil make it harder for roots to absorb water, and make it hazardous for plants. Salinity-resistant plants display morphological, biochemical, and physiological adaptations in an effort to maintain their life cycles. It’s estimated that 50% of cultivated agricultural lands will be under salt stress by 2050 (Shrivastava and Kumar, 2015; Salts of NaCl and Na_2_SO_4_ are the main reasons affecting the salinity of agricultural lands (Pessarakli and Szabolcs, 2010). Germination and early seedling stages are the most susceptible stages to soil salinity (Munns and Gilliham, 2015). By disrupting ionic and osmotic equilibrium, salinity creates stress, which ultimately causes physiological drought in plants. Salt stress causes a number of cellular and metabolic changes such as cellular growth and expansion disruption, plant membrane instability, ion toxicity, altering metabolism, inhibited seed germination, reduced photosynthesis, and reduced shoot, root, and leaf development in various crops (**Table 3**).

**Table 3:** Impacts of salinity stress on some major crops.

Crop	Effect	Reference
Rice	Excessive accumulation of Na+ ion in the root, reduction in the plant root and shoot growth, fresh weight, poor development of spikelets and panicle sterility, and loss of grain yield	Kazemi and Eskandari, 2011; Hussain et al., 2017; Munns, 2002; Hussain et al., 2019
Wheat	Decrease in seed germination, reductions in the growth and development of shoot and roots, leaves, and cells, decreases in ion transfer, gaseous exchange, decrease in the photosynthetic ratio and yield loss	Wahid et al., 2006; Motos et al., 2017; Zhang et al., 2017
Maize	Hampered seed germination, decrease in shoot growth, necrosis	Khodarahmpour et al., 2012; Farooq et al., 2015
Sorghum	Mineral deficiency, ion toxicity, decrease in plant stem yield and photosynthates	Netondo et al., 2004; Almodares et al., 2014
Cotton	Leaf area reduced, reduced plant growth, root and shoot growth, decreases in photosynthetic activity, Fiber quality, metabolic activities, decrease in fiber quality	Muhammad et al., 2018; Hussain et al., 2019
Coconut palm	Reduction in CO_2_ permeability, photosynthetic inhibition,	Gomes and Prado, 2007
*Medicago truncatula*	Damaged Photosystem II, reduction in photosynthesis rate, inhibition of gaseous exchange	Najar et al., 2018

Along with the aforesaid effects, there is a fall observed in osmotic potential which ultimately reduced the uptake of nutrients and water by salinity stressed roots (Jose et al., 2017). Salinity induced stomata closure led to the inhibition of CO_2_ fixation and destruction of photosynthetic pigments (Qados, 2011), which adversely affected the photosynthetic processes, and electron carrier (Sudhir et al., 2005). Salinity stress has a negative impact on plants considering the hormonal level as well as nutrient level. It causes a hormonal imbalance of the ABA, and IAA levels in stressed plants as reported (Wu et al., 2005). Further, salts of NaCl increases concentrations of Na^+^ and Cl^-^ ions which put forward the ionic stress by getting in to competition with essential nutrients such as K^+^, Ca^2+,^ and Mg^2+^ leading a nutrient deficiency condition in plants (Botella et al., 2007). The aforesaid negative implications of NaCl salt will gradually lead to decreasing photosynthetic activity, generation of ROS, and programmed cell deaths (Serrano et al., 1999).

## 3 Response of phytohormones during abiotic stress

Low molecular weight phytohormones are considered to be the most important endogenous compounds having a crucial role in regulating physiological reactions of helps plants to heal in adverse environmental stress condition. (Khan et al., 2013). Reduced seed germination and plant growth have been linked to lower endogenous levels of phytohormones which can further be aggravated by various abiotic stresses (Iqbal et al., 2006). Stress can induce and activate various plant endogenous phytohormonal activities which further help in expression of various beneficial plant genes and proteins (Hamayun et al., 2010). Exogenous phytohormone application has also been proposed as a useful tactic to address various abiotic stresses, such salinity, drought, etc. (Iqbal et al., 2006), and also associated with several studies in reducing the negative impacts of abiotic stressors (Sharma et al., 2013; Iqbal and Ashraf 2013a, b; Amjad et al., 2014). The primary location for auxin production is in the apical meristem of shoots, immature leaves, and seeds. They contribute to phyllotaxis, apical dominance, root formation, embryogenesis, and reaction catalysis. In the molecular mechanism of auxin production, the TRYPTOPHAN AMINOTRANSFERASE OF ARABIDOPSIS (TAA) family, YUCCA gene families are the most important contributors. Majorly *YUC* gene family from which YUC flavin monooxygenases (YUC1, YUC2, YUC4, and YUC6) play essential roles in its auxin biosynthesis and plant development (Cheng et al., 2006).

The main cytokinins found in higher plants are zeatin, isopentenyl adenine, and dihydrozeatin, however zeatin is the most common cytokinin (Kieber and Schaller, 2018). The inhibition of lateral root initiation (Bielach et al., 2012), differentiation of phloem and metaxylem in roots (Bishopp et al., 2011), differentiation of photomorphogenic cells in expanding leaves and shoots (Efroni et al., 2013), and inhibition of leaf senescence are just a few examples of the significant regulatory functions of cytokinins at the tissue and organ levels (Zwack and Rashotte, 2015). The phytohormone has a good control over cell division (Miller et al., 1955), cell homeostasis, and adaptation of plants to climate change (Landrein et al., 2018). ABA is also known as stress hormone as whilst under stress, plants build up ABA, which sets off a reaction to deal with the adverse environment (Mahajan and Tuteja, 2005). It is a signaling molecule for regulation of seed germination and plant growth and development and seed maturation (Yan and Chen, 2016). From seed germination until senescence, the physiological and developmental processes of plants are thought to be significantly regulated by ethylene (Pierik et al., 2006). It plays a part in the regulation of photosynthesis, the metabolism of nutrients and proline and the antioxidant defense mechanism that shields plants from environmental stressors. Numerous studies have shown both benefits as well as negative impacts of the phytohormone. while in corn, *Arabidopsis*, tomato, and grapevines, ethylene and its precursor ACC (1-aminocyclopropane-1-carboxylate) helps to tolerate environmental adversities; in *Cucurbita pepo*, tomato, *Arabidopsis*, and tobacco ethylene claimed its negative impact on plant growth (Lin et al., 2012; Yang et al., 2013; Freitas et al., 2017; Gharbi et al., 2017; Xu et al., 2019; Cebrián et al., 2021). The genetic basis unravels the APETALA 2/ethylene-responsive element binding factor (AP2/ERF) which is a plant specific transcription factor family is an important ethylene biosynthesis factor. It has four major subfamilies: DREB (Dehydration Responsive Element-Binding), ERF (Ethylene-Responsive-Element-Binding protein), AP2 (APETALA2) and RAV (Related to ABI3/VP), and Soloists (few unclassified factors). These subfamilies act as crucial regulators in a variety of biological and physiological processes, including signal transduction, regulator of plant morphogenesis, stress-response mechanisms, and metabolic activities (Li et al., 2020). Gibberellins (GA) are growth regulators that are particularly effective for seed germination, stem lengthening, enlarging fruit, and inducing blooming (Camara et al., 2018). Gibberellins’ main function is to promote cell elongation, which in turn promotes cell division, accelerating both the vegetative and reproductive stage of plant growth (Colebrook et al., 2014; Kang et al., 2014). Exogenous GA treatment has also several benefits like it promotes early and large number sprouting in potato tuber (Alexopoulos et al., 2017), further it can improve the amount of viable seeds and antioxidant enzyme activity, increases the weight of individual fruits (Zang et al., 2016). Among the other phytohormones, brassinosteroid (BR) which was initially discovered in pollen of *Brassica napus* (Saini et al., 2015) was reported to be involved in root extension, maintenance of meristem size, initiation of lateral roots, creation of root hairs, mycorrhiza, and nodule formation (Mc Guinness et al., 2019; Wei and Li, 2016). Further during stress condition, crops like maize, soybean and banana are benefitted from methyl jasmonic acid in terms of increasing photosynthetic rate, grain yield, and drought tolerance (Anjum et al., 2016; Yu et al., 2019) Stress responses are essentially driven differently by different phytohormones and their crosstalk, that leads to transcriptional reprogramming in plants’ response. The pivotal roles of phytohormones can be manipulated for mitigating the effect of the stressor.

## 4 Response of nutrients during abiotic stress

All the seventeen essential nutrients of plants are more or less responsible for abiotic stress alleviation in their own way. The most important plant nutrients, nitrogen (N), have an impact on physiology, growth, the reduction of biotic and abiotic stress, and structural integrity (Karim et al., 2016). However, it has a significant impact on crop plants’ ability to effectively use solar energy, increase photosynthetic activity, and synthesize chlorophyll (Waraich et al., 2011). Phosphorus not also improves root architecture and proliferation in the soil even in soil drying conditions, but also stimulates root volume and hydraulic conductivity (Tariq et al.,2017). The modulation of numerous morphological, physiological, and biochemical processes by phosphorous within the plant system helps them to withstand stress better. Plant growth and development under stress are strongly and positively correlated with the use of phosphoric fertilizers. Ge et al. (2012) reported that potassium is another crucial nutrient for many fundamental physiological and metabolic processes including photosynthesis, stomatal control, photosynthesizing, carbohydrate metabolism, preservation of cell turgidity, enzyme activations, etc. Potassium is also essential for improving crops’ tolerance to various abiotic stresses (Danial et al., 2010).

**Figure 1:**
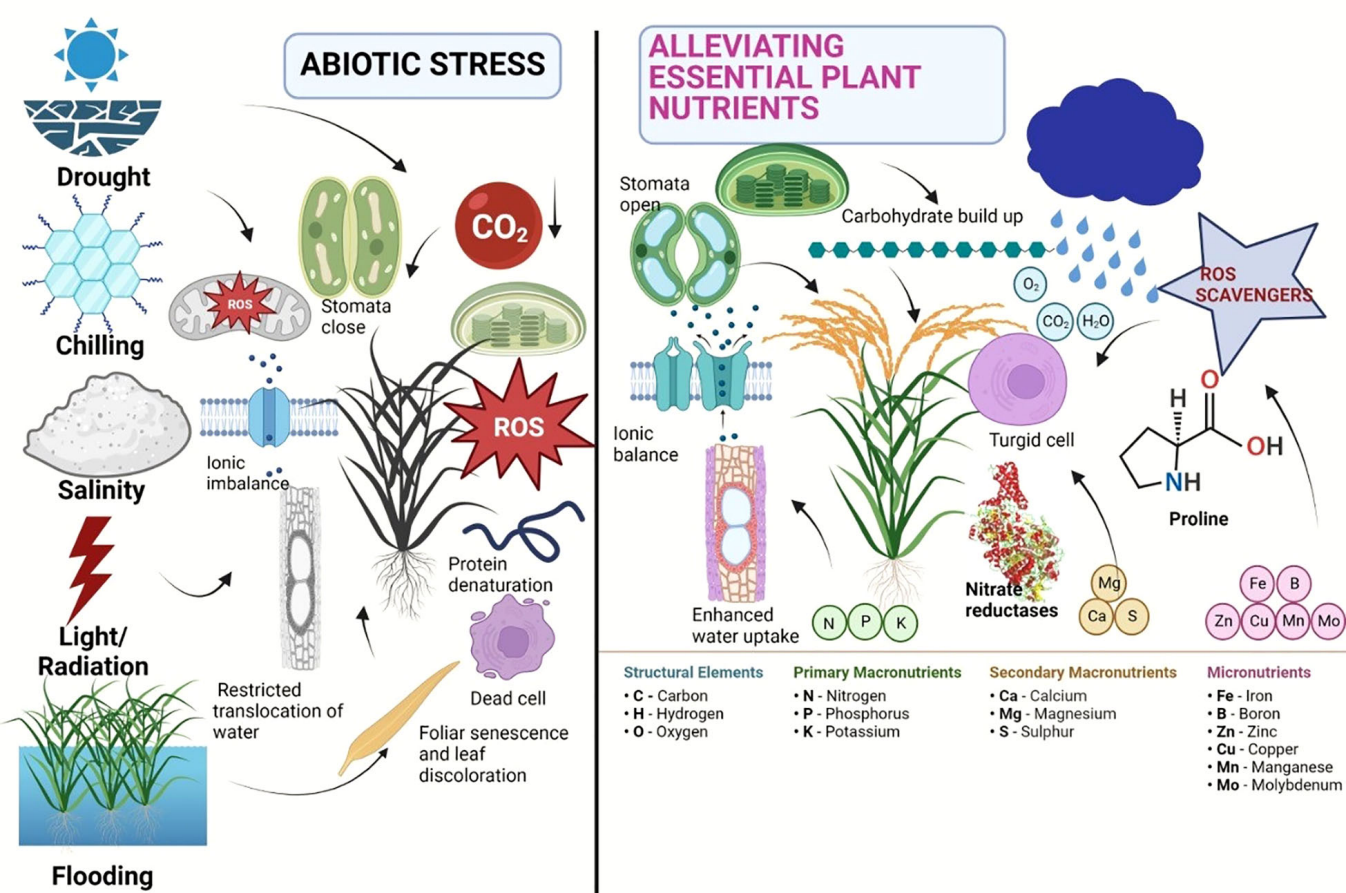
Negative impacts of abiotic stress and their alleviation using plant nutrients in plant.

Calcium (Ca), an important secondary nutrient, acts as a signaling molecule in a number of physiological and biochemical processes that are necessary for a plant to develop stress tolerance (Ahmad et al., 2015). Magnesium (Mg) is essential for the conformational stabilization of macromolecules such as nucleic acids, proteins, cell membranes, and walls and is a structural component of the ribosome (Marschner, 2012). Its absence can have an impact on photosynthesis because it is a crucial element of the chloroplast, which controls photosynthetic activity. In the abiotic stress response, cellular acclimatization, and adaptability to challenging circumstances, sulfur performs protective roles (Cao et al., 2014). According to reports, an exogenous dose of sulfur increases crop productivity while maintaining regular metabolic processes that enable plants survive in harsh settings (Hasanuzzaman et al., 2013). The micronutrients like boron, zinc, iron, and copper reduce environmental stress through a variety of mechanisms, including glucose metabolism and transport, production of cellular integuments, preservation of membrane integrity, and activation of numerous enzymes. The structural role of selenium (Se) in the synthesis of glutathione peroxidase (GPX), which protects plants from the damaging effects of ROS, is also well documented (Lobanov et al., 2008). An adequate supply of Zn shields plants from the damaging effects of heat stress because it plays a significant role in maintaining membrane permeability (Peck and McDonald, 2010). The plant nutrients can be very much effective similar to the phytohormones for alleviation of various negative impacts of abiotic stresses. A brief account of abiotic stress alleviation using plant nutrients has been depicted in **Figure 1**. It is observed that in response to several abiotic stresses, major nutrients like N can enhance the photosynthesis of plant, phosphorus can be able to produce proliferate and strong root system, calcium can enhance the membrane stability and cellular integrity in plant, the micronutrients can able to regulate the cellular activity and mitigate abiotic stress by activating numerous enzyme and selenium can protect the plant from ROS activities.

## 5 Phytohormones and their effect on abiotic stress

During abiotic stress, it was observed that the phytohormones levels are altered; majorly ABA and ethylene level enhanced along with reduction of auxin and cytokinin are seen in a number of crops. The phytohormone works both in the response to stress as well as works for alleviation of stress. Both endogenous and exogenous level of phytohormones is showing equal importance in alleviation by regulating the internal and external stimuli in plants. The genes responsible for the phytohormone level regulation are activated and their upregulation can be helpful for enhancing stress tolerance in plant. When plants are affected by several stresses, especially water deficit, plant hormones play vital roles in their growth and development (Raza et al., 2021). Several plant growth regulators, including salicylic acid, gibberellins, auxins, cytokinin, and abscisic acid, have reacted to drought (Chen et al., 2019). Phytohormones regulate internal and external stimuli, as well as signal transduction pathways, in addition to stress responses. Water logging or flood is a major constraint in low land conditions, the use of phytohormones signaling pathway can lead to a better way to alleviate the stress and achieve higher yield. Cold stress is a major problem in tropical and subtropical crops, whereas heat stress in temperate crops hampers crop production and productivity. It was observed that endogenous phytohormones level like gibberellic acid, brassinosteroids, cytokinins, abscisic acid, salicylic acid, jasmonic acid, and, auxin modified and regulates plant growth. A number of genes are activated during the exogenous application of plant hormones as a result tolerance can be created in the plants. So, studies on gene regulation and translation mediated by phytohormones can unlock a new way to recover low-temperature stress in plants.

### 5.1 Effect of auxin on stress

On exposure to drought, the plasticity of the plant root is affected that is regulated by the auxin. Auxin buildup in the root system reduces daytime and nocturnal water use and modifies hydraulic characteristics to allow the expression of water-saving features in wheat, maize, and sorghum yields during droughts. (Shao et al., 2017; Li et al, 2012; Rama Reddy, 2014). The exogenous application of auxins has shown to be effective in managing drought stress in plants. Indole-3-acetic acid (IAA) is the most common plant hormone of the auxin class and is mainly synthesized from the amino acid tryptophan (Trp). IAA triggers the activation of other stress-responsive hormones as well as the production of ROS. ROS production molds several physiological processes in a plant in response to water deficit stress. Discovery and characterization of numerous auxin-responsive genes in a number of plant species including rice, soybean, and Arabidopsis has paved the way for exploiting the genes to induce stress response (Hagen and Guilfoyle, 2002). A membrane-bound transcription factor NTM2 was used for auxin signaling controls for seed germination in salinity stress (Jung and Park 2011; Park et al.,2011). The number of genes like TRYPTOPHAN AMINOTRANSFERASE OF ARABIDOPSIS (TAA) family, YUCCA gene family is the most important contributors to auxin biosynthesis (Cheng et al., 2006) that controls several metabolic activities in the drought-affected crop plants. **Table 4** highlighted the impact of auxin-linked genes on the stress response.

**Table 4:** Impact of auxin-linked gene on stress response.

Crop	Gene	Physiological impact	Reference
Transgenic rice	Expression of auxin-coding genes *OsIAA6*	Tillering behavior	Jung et al., 2015
Transgenic poplar and potato	Overexpression of YUC6	Faster shoot growth and retarded main root development with enhanced root hair formation, reduced levels of ROS production, higher photosystem II efficiency, and less membrane permeability	Ke et al., 2015
Tomatoes	Auxin-responsive genes (WRKY108715, MYB14, DREB4, and bZIP 107)	Increased root density and growth, maintained chlorophyll content, and increased soluble sugar content	Bouzroud et al., 2018; Zhang et al., 2020
White clover	Up-regulated auxin responsive genes (GH3.1, GH3.9, IAA8), drought stress-responsive genes (bZIP11, DREB2, MYB14, MYB48, WRKY2, WRKY56, WRKY108715 and RD22), and down-regulated leaf senescence genes (SAG101 and SAG102)	Increased stem dry weight, chlorophyll content, delayed senescence	Zhang et al., 2020
Arabidopsis	Expression of auxin responsive IAA5/6/19	Maintained level of glucosinolates (GLS), regulation of stomatal closure and ROS production	Salehin et al., 2019
Wheat	TAA family gene TaTAR2.1-3A overexpression	Increased grain yield under various nitrogen supply levels, high lateral root branching	Shao et al., 2017
Sorghum	IAA-amido synthetase gene GH3.5	Stay green	Rama Reddy et al., 2014
Tobacco seedlings	Initial elevated DR5: GUS gene expression levels and later decreased expression levels	Lateral root branching	Wang et al., 2018a

Auxins have significant involvement in temperature-related stress. Shibasaki et al. (2009) performed a direct transport assay using an auxin-responsive marker (*IAA2-GUS*) on cold stress and concluded that the intracellular auxin efflux carriers are inhibited in plants due to cold stress. In high temperature, the plant mainly suffers due to reduction of pollen viability in major crop, affecting seed set and eventually reducing yield. In crops like wheat and barley, it was observed that the initial pollen development stage is majorly hampered due to the reduction of pollen auxin concentration at high temperatures (Tadashi et al., 2010). They concluded that tissue-specific auxin concentration reduction can lead to pollen abortion during high-temperature stress. An analogous study was reported by Zhang et al. (2018) through an experiment in rice by exposing the rice spikelet to high temperature which drastically reduced the spikelet fertility and mitigated its effect by the application of NAA (1-naphthaleneacetic acid). Through the application of NAA in rice crops, auxin concentration was increased and leads to the proper development of pollen tube growth, elongation in the pistil, stylar length of the flower, and ultimately the pollen behaves normally under high temperatures leading to proper pollination and fertilization. In wheat, the effect of the exogenous application of auxin was estimated under heat stress conditions and found that the application of 1 µM of IAA can enhance higher grain number and yield by 6% – 8% under heat stress conditions (Abeysingha et al., 2021).

### 5.2 Effect of abscisic acid (ABA) on stress

Abscisic acid is a signaling molecule in plants in responses to stress conditions and noted as second group of phytohormone. ABA is a 15-carbon atom compound that belongs to a group of metabolites, known as isoprenoids or terpenoids, which are synthesized in the plastids (Xiong and Zhu, 2003; De Ollas et al., 2013). Under optimal conditions, ABA is expressed at low concentrations in plants (Parveen et al., 2021) and the concentration increases with the signal of stress to plants. In drought conditions, ABA alteration of guard cell ion transport regulates stomatal opening that reduces water loss (Kim et al., 2010). Upon exposure to drought stress, ABA is synthesized in roots and translocated to leaves wherein mesophyll cells are the predominant location of ABA synthesis (McAdam and Brodribb, 2018). Biosynthesis of ABA triggers drought adaptation mechanisms in the plants such as growth retardation, stomata closure and activation of several drought-responsive genes (Qi et al., 2018; Wilkinson and Davies, 2010). ABA regulates turgor by decreasing transpiration as well as by increasing water influx into roots (. Root-specific ABA signaling helps in balanced root growth toward soil exploration which regulates the transpiration and increases water influx into roots (Glinka and Reinhold, 1971, Duan et al., 2013). Considering the molecular activities of the phytohormone, in drought tolerant transgenic *Arabidopsis* overexpression of IbARF5 gene up-regulates the ABA biosynthetic genes (IbZEP, IbNCED, and IbABA2) was reported (Kang et al., 2018). Various transcription factors such as DREB2A/2B, AREB1, RD22BP1, and MYC/MYB are known to be the regulator of the ABA-responsive gene expression (Tuteja, 2007). SAPK2 ((Stress-Activated Protein Kinase) of SnRK2s (Sucrose nonfermenting1–Related protein Kinase 2) family which is an important ABA regulator, upregulates the expression of several drought responsive genes, including OsLEA3, OsOREB1, OsRab16b, OsRab21, and OsbZIP23 (Lou et al., 2017). From the aforesaid reported studies, it has been clear about the roles of endogenous ABA regulator genes and their role in the drought alleviation. Similarly, the exogenous application of ABA during drought in maize seedlings can also play role in activating antioxidant enzymes which in molecular basis regulates expression of ASR1, and endogenous ABA level, as well as reduce oxidative damage (Yao et al., 2019).

Similar to drought, salt-responsive genes’ expression is also known to be regulated by both endogenous and exogenous ABA-mediated signaling when the soil is affected by salinity (Wang et al., 2001, Narusaka et al., 2003). Zhang et al. (2006) found a proportionate link between plants’ exposure to salinity and their ABA content. Both endogenous ABA and its exogenous application demonstrate a critical role in preserving ionic balance in plants, as shown by their ability to prevent chloride toxicity in citrus leaves, avoid Na^+^ and Cl^-^, and maintain K^+^/Na^+^ ratio response in rice, K^+^ and Ca ^+^ homeostasis (Gomez et al., 2002; Bohra et al., 1995; Gurmani et al., 2013). In addition to stomatal regulations, this phytohormone also aids in the osmoprotectants like proline (Iqbal et al., 2014) and dehydrins in response to ROS production during salinity-induced dehydration (Szabados and Savoure, 2009; Kim and Wang 2010; Hara 2010; Gurmani et al. 2013). Sripinyowanich et al. (2013) reported that, the expression of the OsP5CS1 gene increased proline accumulation and increased the survival rate (20%) of *Indica* rice seedlings by exogenous application of 100 M ABA. Shi and Zhu (2002) noted that ABA induced AtNHX1 expression in barley in response to salt stress. Keskin et al. (2010) stated that, ABA treatment caused quicker expression of MAPK4-like genes (TIP1 and GLP1) in wheat crops under salinity. Apart from drought and salinity, the exogenous application of abscisic acid (ABA) and ethylene also plays role in controlling other abiotic stresses. In green under greenhouse conditions the above hormones inhibit the suppression of photosynthesis in waterlogging by rejuvenating several factors like photosynthetic rate, transpiration rate, stomatal conductance, chlorophyll content and leaf water potential (Ahmed et al., 2002).

In case of cold stress, the effect of ABA application was studied on bermudagrass at 4 ^0^C with application of 100 μM ABA which showed increased levels of chlorophyll content, maintained cell membrane stability, improved the performance of photosystem II, and altered expression of ABA or cold-related genes, including ABF1, CBF1, and LEA developing cold resistance in the grass (Huang et al., 2017). When wheat was exposed to low temperatures (0°C, −10°C, −20°C, and −25°C) application of exogenous ABA decreases the amount of H_2_O_2_ and relative conductivity (Jing et al., 2020). ABA was found to enhance cold tolerance in both leaves and rhizomes at −10°C and −20°C by increasing ROS production (Jing et al., 2020). In the same way, the ABA application was studied for its effect on heat tolerance in two rice germplasm lines. Li et al. (2020) used rice germplasm lines having flat leaves called wild type (WT) and others having rolling leaves with high-temperature sensitivity (*hts*) lines exposed to high temperature. The high-temperature lines showed a higher respiration rate with high tissue temperature and lower transpiration rate and stomatal conductivity but the WT line showed increased carbohydrate content, dry matter increased production of heat shock proteins (HSP71.1 and HSP24.5) under high-temperature stress. Through ABA application in these two lines, it was observed that thermo-tolerance was increased in the wild type but tolerance was reduced in *hts* plants (Li et al., 2020).

### 5.3 Effect of cytokinins on stress

Many drought-related processes are mediated by the stress hormone (ABA) as well as cytokinins (CKs). When plants are under drought stress, their CK content falls, and this increase in ABA responses causes the stomata to close and impede photosynthesis (Rivero et al., 2010). Stomatal conductance and transpiration are increased by CKs’ ability to keep the stomata open (Lechowski, 1997). These CKs and ABA alterations brought on by stress encourage early leaf senescence and hormonal adjustments that cause leaf abscission, which results in a smaller canopy and less water loss. Pospisilova et al. (2000) observed that the expression of a cytokinins biosynthetic gene isopentenyltransferase (IPT), catalyzes the rate-limiting step in CK synthesis. Overexpression of IPT enhances the antioxidant system activity and increases drought tolerance by improving root growth in plant (Xu et al., 2016). Hormones like cytokinin enhance primary root growth in Arabidopsis by giving positive signaling to plant (Naulin et al., 2020). I In transgenic barley (*Hordeum vulgare*) and in tobacco it was observed that root specific reduction of cytolinin led to the enlarged root system under stress situations (Werner et al., 2010; Pospíšilová et al., 2016). It has also been shown that the increased transcriptional level of *CKX* genes and/or CKX activity was due to the exogenous application of cytokinin. It was observed that due to oxidase/dehydrogenase (CKX) which catalyzes CK and the overexpression and breakdown of CKX in Arabidopsis carried out as a result endogenous CK contents is decreased in plant (Werner et al., 2010). The abnormal expression of *CKX* in barley *via* maize *β-glucosidase, a* mild root-specific promoter has also been found to alter root architecture leading to lignification of the root tissue as well as activation of flavonoids biosynthesis (Vojta et al., 2016). Plant shows a higher level of accumulation of CK in root tissues due to a decrease in the activity of CKX, during drought stress (Havlova et al., 2008). The plant growth rate was slow down and elevate the content of protective compounds due to overexpression of CKX, which finally gives rise to increased drought tolerance in *Arabidopsis*, tobacco, and barley (Macková et al., 2013; Nishiyama et al., 2011; Pospíšilová et al., 2016).

Salt-sensitive plants’ development was negatively impacted by salinity by lowering CK levels, indicating genotypic specificity (Kuiper et al., 1989). After being exposed to salinity, the amounts of CKs such as zeatin (Z), zeatin riboside (ZR), and isopentenyl adenine (iP) in the shoots and roots of barley cultivars drastically decreased (Kuiper et al., 1990). The negative effects of salt on plant growth are also known to be mitigated by CKs (Barciszewski et al., 2000; Fahad et al., 2014). Plant resistance to salt stress was reported to enhance with seed primingB with CKs (Iqbal et al., 2006). Iqbal et al. (2006) reported that CKs operate as ABA antagonists and IAA antagonists/synergists in a variety of plant processes and assist reduce salinity stress (Iqbal et al., 2014). Under exogenous application of CKs, it enhanced salt resistance *via* increased proline levels in brinjal (Wu et al., 2013). Plant hormones, particularly CKs, control the expression of a large number of stress-induced genes. Merchan et al. (2007) reported that the changes in osmotic circumstances also affect the expression of CKs receptor genes, showing that these receptors may have a similar function in the osmotic stress response despite the lack of a clear mechanism.

In waxy corn, exogenous application of 6-benzyl adenine (BA) in water logging conditions noted that not-treated plants showed chlorosis and necrosis in leaves, inhibiting growth and leading to the accumulation of O_2_, H_2_O_2_, and MDA-like reactive oxygen species (ROS) but in treated plants, the reduction of ROS accumulation and increase of enzyme activities like ascorbate peroxidase, glutathione reductase, dehydroascorbate reductase, and monodehydroascorbate reductase (Wang et al., 2021). Hence, the application of exogenous BA can alleviate water-logging-induced damage and improve water logging tolerance in waxy corn *via* the activation of the AsA-GSH cycle system and the elimination of ROS. The application of BA in waterlogged maize crops showed enhanced grain filling by improving grain weight and volume, which was beneficial to yield increase as compared to the untreated plant (Baizhao et al., 2019). It was recorded that the application of exogenous BA alleviated endogenous hormone levels of IAA, zeatin, and GA_3,_ and at the same time, ABA content was decreased during grain-filling periods of waterlogged summer maize. The foliar application of CK and GA_3_ under waterlogged conditions revealed that growth and biomass were enhanced, which was associated with increased levels of photosynthetic rate and pigments in the plant (Islam et al., 2022). It was reported that the accumulation of ROS and malondialdehyde levels is reduced during the water logging condition by application of CK and GA_3_. Therefore, a better osmotic adjustment was carried out through proline and TSS level improvement in plants. Both CK and GA_3_ were effective in water-stressed plants, however, CK was considered more effective than GA_3_ (Islam et al., 2022). Prerostova et al. (2021) identified two genes which to be associated with cytokinin metabolism in plant, i.e., CK biosynthetic gene isopentenyl transferase (DEX: IPT) and CK degradation gene HvCKX2 (DEX: CKX). They observed that plants containing the DEX: IPT gene showed better stress tolerance with increased production of CK and SA levels in shoots and also auxin in the apex. At the same time plant containing the DEX: CKX gene and control plants showed weaker stress tolerance with lowered levels of CKs and auxins in cold conditions.

### 5.4 Effect of ethylene on stress

Ethylene (ET) has a significant role in fruit softening along with a vital role in mitigating the harmful impact of stress conditions due to abiotic factors (Pech et al., 2018; Wang et al., 2020). In diverse range of abiotic and biotic stress condition it was found that ET has a major role in nodule formation and nodule signaling (Khalid et al., 2017). Furthermore, it also enhances root emergence from nodal region which leads to retardation in development of nodal root and ultimately give rise to a negative effect on root-lodging resistance in *Zea mays* (Shi et al., 2019). Drought induces ethylene synthesis in shoots, by up-regulating the synthesis and xylem transport from roots to shoots of the ethylene precursor ACCs (Sobeih et al., 2004). It was found from research that adventitious root initiation sites in *Arabidopsis* hypocotyls are controlled by ethylene (Rasmussen et al., 2017). The overexpression of ethylene response factor such as GmERF3 of AP2/ERF gene family, leads to improvement in proline content, soluble sugar, and decreases in the accumulation of malondialdehyde to improve drought tolerance in the tobacco plant (Zhai et al., 2017). Further, SlERF5 of the aforesaid transcription family in over-expressing transgenic tomato plants resulted in high tolerance against drought (Pan et al., 2012). It was also found that gene 269 AP2/EREBP in cotton showed water stress response in plant (Liu and Zhang, 2017). Ethylene application response was also studied under water logging stress in soybean. It was noted that after the application of ETP (ETP; donor source of ethylene) in soyabean under water logging stress, the chlorophyll content significantly enhanced, and also cellular gibberellic acid is increased in the treated plant as compared to untreated plants (Yoonha et al., 2018). The amino acid content was also found appreciably higher in ETP-applied soybean plants than in the control. Several adventitious roots were induced in the plant after ETP application which enhance the root surface area and considerably amplified the expressions of glutathione transferases which that control ROS under water stress (Yoonha et al., 2018).

In the case of *Arabidopsis thaliana*, it was observed that freezing tolerance decreases by the introduction of the *ethylene overproducer1* gene and by the application of the ethylene precursor 1-aminocyclopropane-1-carboxylic acid but the freezing tolerance enhanced when ethylene biosynthesis inhibitor amino-ethoxyvinylglycine was applied (Shi et al., 2012). Shi et al. (2020) thus suggested from their research that ethylene can negatively regulates cold signaling through the direct transcriptional control of cold-regulated *CBFs* and type-A ARR genes. Sun et al. (2016) found a positive correlation between ethylene (ET) and cold stress was studied in grapevine. The treatment of exogenous 1-aminocyclopropane-1-carboxylate a form of ethylene was able to mitigate the cold stress in crops compared to the application of ET biosynthesis inhibitor amino-ethoxyvinylglycine which reduced the cold tolerance of grapevine. It was also observed that overexpression of gene ‘VaERF057’ enhances cold tolerance in *Arabidopsis* and ethylene is associated with the signaling of this gene. Thus, the research concluded that ET positively regulates cold tolerance in grapevine by regulating the expression of VaERF057 gene associated with cold tolerance (Sun et al., 2016). Wang et al. (2021) reported in case of apple seedlings, when treated with 1-aminocyclopropane-1-carboxylate (an ethylene precursor) and amino-ethoxyvinylglycine (an ethylene biosynthesis inhibitor), it was observed that the cold tolerance was increased and decreased respectively in the crop. They reported that during low-temperature treatment, ethylene level enhanced which leads to the over expression of MdERF1B significantly, increasing the cold tolerance of apple planting materials (seedlings and calli) as well as in *Arabidopsis* seedlings by mediating ethylene signaling pathway. Furthermore, molecular analysis proved that MdERF1B interacted with the promoters of two ethylene biosynthesis genes, i.e., MdACO1 and MdERF3. Wang et al. (2021) result thus concludes that MdERF1B–MdCIbHLH1 is a potential regulatory pathway that integrates the cold and ethylene signaling pathways in apples by up-regulating ethylene production under cold stress. While under high-temperature stress or heat stress ethylene is found to affect the pollen viability and sterility in plants similar to the auxins. In research conducted by Jegadeesan et al. (2018), it was observed that tomato pollen sterility can be overcome by the application of ethylene hormone (ethephon) during heat stress conditions. A protein analysis conducted during the study showed pollen development was hampered during heat stress due to the degradation of some proteins responsible for pollen development, pollen tube germination, and tube growth under the pistil surface. Jegadeesan et al. (2021) reported that ethylene hormone had a positive impact on pollen viability and germination, and the ability to increase the overproduction of heat tolerance genes like *SlHSP17*, SlHSP*101*, *SlMBF1* in tomatoes, when applied exogenously reducing the harmful effects of heat stress in due course. Another study in wheat showed ethylene again plays a vital role by regulating the biosynthesis of proline and modifying the antioxidative mechanism under heat stress. Application of 200 µL of ethephon and 50mM of proline showed improved tolerance of wheat in heat stress by activation of defense mechanism and protecting the photosynthetic pigment by enhancing the photosynthetic gene expression in crops (Sehar et al., 2022).

### 5.5 Effect of gibberellins on stress

On drought stress conditions, down regulation of GA could be a major target in making drought-tolerant plants. Nir et al. (2014) reported that the transgenic plants with the lower GA level tend to produce high stomatal intensity, lower stomatal conductance, and smaller leaves, which reduces the transpiration rate in stress. Further, the overexpression of SlDREB of the AP2/ERF family down-regulates GA biosynthetic genes in tomatoes. In tomato internode elongation and leaf expansion is reduced as a result of lower GA level in plant which ultimately create drought tolerance mechanism in plant (Li et al., 2012). Further studies confirmed water deficiency leads to downregulation of GA biosynthesis genes GA20 oxidase1 (GA20ox1) and GA20ox2 and induce the GA deactivating gene GA2ox7 in guard cells and leaf tissue, resulting in reduced levels of bioactive Gas in tomato (Shohat et al., 2021). Moreover, the over-expression of another transcription factor PtGA2ox1 decreases the GA level in the roots, stems, and leaves of the tobacco plant to promote drought tolerance (Zhong et al., 2014). In addition to maintaining protein and RNA levels, higher water level was also credited with GA’s beneficial effects under salinity stress (Yamaguchi 2008). Maggio et al. (2010) reported that the application of GA to tomato plants reduced stomatal resistance and increased plant water usage effectiveness at lower salinity levels. Under salinity, the root and leaf cell nitrogen and magnesium are increased due to GA application (Tuna et al., 2008). Multiple factors, including an increase in reducing protein synthesis, activity of enzymatic antioxidants, sugars, and decreased activity of ribonuclease and polyphenol oxidase, contributed to GA3’s beneficial effects on salt-stressed mung bean seedlings (Mohammed, 2007). Modulation of ions absorption and partitioning (inside shoots and roots) as well as hormonal homeostasis brought on by GA3 priming under salinity (Fahad et al., 2014). Through changed GA levels, the seed germination rate is enhanced due to the salt-inducible DDF1 gene (dwarf and delayed flowering 1) in high saline stress condition.

The applications of gibberellins in soybean plants found to reduce chlorophyll damage and also enhance the endogenous level of GA1 and GA4, and jasmonic acid in the plant along with the reduced level of ABA under flooding conditions (Muhammad et al., 2018). The research reveals that exogenous application of GAs during short-term waterlogging could enhance the transcriptional pathways and biochemicals which are majorly needed for maintaining plant growth during stress. Calvin et al. (2019) reported that the application of GA3 (200 ppm) in combination with salicylic acid (150ppm) on the soybean plant provides better mitigation effects by improving the number of pod and seed, chlorophyll content in waterlogged conditions. Gibberellins were found to be extremely sensitive towards cold stress and several GA metabolic genes, GA3ox1, GA20ox1, and GA2ox1 were found to be activated during cold temperatures (Ding et al., 2015). GA3 treatment has also improved fruit storage under low temperatures by decreasing malondialdehyde content and electrolyte leakage, increasing proline content and improving antioxidant enzyme activities as compared to untreated conditions (Ding et al., 2015). Shashibhusan et al. (2021) observed that pre-treatment of plants with 1gm, 2gm, and 3 gm of GA3 promotes plant growth and other yield-attributing traits in cold stress conditions in rice. Whereas, GA3 was found to have no direct role in heat tolerance but rather be associated with cell expansion gene activation and also positively affect the test weight of the seed in wheat (Nagar et al., 2021). They also noted that the application of paclobutrazol showed a thermo-tolerance effect rather than GA3 biosynthesis inhibition in wheat. Guo et al. (2022) suggested that gibberellins can mitigate the effects of heat stress response in plants by providing evidence obtained in tomatoes. They concluded from the result that exogenous application of gibberellic acid (GA_3_) of 75 mg/L can mitigate heat stress by improving the plant growth, morphology, and physiological characteristics of tomatoes.

### 5.6 Effect of brassinosteroids on stress

Under stress condition, BRs increase Rubisco and the water usage efficiency of leaves hence improving CO2 assimilation and leaf water economy (Farooq et al., 2009). Several studies have also revealed that brassinosteroids s play a beneficial function in drought-stressed *Brassica napus*, wheat and *Arabidopsis* (Kagale et al., 2007). Exogenous 24-epibrassinolide treatment raises BRs content while lowering ABA and ROS levels, which further aids in increasing stomatal hole for water stress resistance (Nie et al., 2019; Tanveer et al., 2019). Unraveling the molecular basis of BRs control, three WRKY transcription factors—WRKY46, WRKY54, and WRKY70—have been identified as crucial signaling components that play oppositely positive and negative roles in BRs-regulated growth and drought responses (Chen et al., 2017). It was reported that the overexpression of a BRs biosynthetic gene AtDWF4, isolated from *Arabidopsis* in applied in transgenic *Brassica napus* results in improved drought tolerance (Sahni et al., 2016). BRs along with ABA showed a major role in drought stress in plants.

The negative effects of salt on plant growth performance are also known to be mitigated by BRs (Zhu 2002; Krishna 2003; Zhang et al., 2007; Kartal et al., 2009; Wang et al., 2011). By restoring pigment levels and elevating nitrate reductase activity, application of BRs through exogenous application reduced the negative effects of salt stress on root elongation, seed germination, and subsequent growth of rice (Anuradha and Rao 2001). Krishna (2003) found that barley leaf segments pre-incubating with BRs prior to exposure to salinity was successful in minimizing the cells’ ultra-structures, such as their nucleus and chloroplasts. Under salinity, treatment of seed with BL considerably improved the accumulation of dry matter and antioxidant enzyme activity in lucerne (Zhang et al., 2007). In rice, Arabidopsis, and brassica, treatment with 24-epibrassinolide significantly increased seed germination, seedling growth, antioxidant system, and proline content, while reducing lipid peroxidation under salinity stress (Ozdemir et al., 2004, Kagale et al., 2007, Divi et al., 2010).

### 5.7 Effect of jasmonate on stress

Jasmonic acid (JA) encourages plant water uptake and methyl JA encourages increased osmoprotectant and compatible solute accumulation to increase chlorophyll content, antioxidant activity, and leaf gas exchange to trigger stomatal closure and improved water usage efficiency (S’anchez-Romera et al., 2014). There were negative impacts during drought stress; it also modifies polyamine and endogenous phytohormones (Xiong et al., 2020). It has been shown that exogenous administration of 0.5 mM methyl JA can preserve wheat growth and output during water deficit stress (Anjum et al., 2016). The application of 10 M methyl JA to sugar beet decreases the negative impacts of severe drought (Fugate et al., 2018). Kang et al. (2005) reported the comparison of salt-sensitive and tolerant rice cultivars and observed that salt-tolerant rice cultivars have a much higher concentration of JA. A critical component of the barley response to salt was thought to be the induction of JA-responsive genes (Walia et al., 2006). Endogenous JA contents in barley leaf segments that were subjected to sorbitol or mannitol osmotic stress increased significantly (Kramell et al., 2000).

JA is considered to have a major role in alleviating heat and light stress damage in the plant. A study conducted in the *Arabidopsis* crop showed a combination of high light and high heat (HL+HS) stress causing major damage to photosynthetic pigments and reducing the D1 protein level in plants with the same time accumulation of jasmonic acid that may provide tolerance in plant (Balfagón et al., 2019). They found that the plant deficient in jasmonic acid is highly sensitive to heat and light stress. Convergent study was conducted in Ryegrass; a temperate grass is sensitive to high temperatures. In this study impact of jasmonic acid on ryegrass was studied, it was observed that methyl jasmonic acid (MeJA) has a positive effect on augmenting tolerance in plants to a high temperature by altering the antioxidant defense mechanism, decreasing chlorophyll loss due to heat, maintaining good water balance in plant and lowering electrolyte leakage in the crop (Su et al., 2021). Along with that also the plant oxidize activity was enhanced by exogenous MeJA treatment which can increase the scavenging ability of ROS produced during heat stress and leads to alleviating the oxidative damage caused by heat stress and production of more heat shock proteins may be expressed in the plant during heat stress condition. (Su et al., 2021).

### 5.8 Effect of salicylates on stress

A phytohormone called SA is produced by chloroplasts (Dempsey and Klessig, 2017). According to reports, SA treatments sustain the cell’s turgor pressure by increase the amount of osmolyte and proline in the root and shoot without affecting the other metabolic processes. Further, when SA is applied exogenously to canola, it increases the number of pods and seed output and is also involved in cell division and expansion (Keshavarz and Sanavy, 2018). Additionally, its use on marigolds under drought stress boosts bioproduction, enhances a number of physiological processes, and lessens the detrimental effects of water stress (Abbas et al., 2019). When crop plants under drought stress, such as wheat, saffron, and *Brassica rapa*, are exposed to it, SA activates nonenzymatic defensive mechanisms like sugar accumulation for energy saving and osmoregulation and lowers their malondialdehyde and free radical contents (Chavoushi et al., 2019; Ilyas et al., 2017). Through redox homeostasis and proline metabolism in agricultural plants, SA treatment increases drought-stress resistance (Chavoushi et al., 2019; Ilyas et al., 2017); La et al., 2019). By accumulating endogenous SA, the Arabidopsis loss of function lines cpr5 and acd6 demonstrated a drought tolerance mechanism (Miura et al., 2013). It has been revealed that in Arabidopsis, the SIZ1-mediated buildup of endogenous SA improves drought tolerance and encourages stomatal closure (Miura et al., 2013). It was observed that the osmolyte content in the vegetative phase of barley, safflower and corn has been increased by triggering multiple defense mechanisms, along with the antioxidant system through exogenous administration of SA which improve drought tolerance in those plants (Abdelaal et al., 2020; Chavoushi et al., 2019). Thus, a potential transgenic strategy for making plants resistant to drought would be to target genes involved in triggering the effect of drought resistance in response to the exogenous administration of SA.

SA’s salt-ameliorating effects have also been widely reported in various crops including bean (Azooz 2009), wheat (Sakhabutdinova et al., 2003), barley (El-Tayeb, 2005), and mung bean (Khan et al., 2010; Syeed et al., 2011). Another study indicated that SA treatment of salt-stressed maize and mustard increased their ability to tolerate salt by speeding their photosynthesis and carbohydrate metabolism (Khodary, 2004; Nazar et al., 2011). Bastam et al. (2013) applied an exogenous treatment of SA to enhance the salt tolerance of pistachio seedlings. Palma et al. (2009) reported that under salinity stress, SA activates the antioxidant systems and is also attributed to the buildup of suitable solutes like proline and glycine betaine (Nazar et al., 2011). In addition, plants treated with SA showed reduced levels of membrane permeability and lipid peroxidation, which were otherwise rather significant under salinity (Horvath et al., 2007). Salicylic acid (SA) application was studied under waterlogging conditions in wheat crops revealing that lateral roots development was enhanced along with the emergence of surface adventitious roots which originate from the basal stem nodes of wheat, but root elongation was hindered, leading to the development of a shallow root system able to survive in water logging condition. (E scholar encyclopedia, 2022). The effects of salicylic acid become more apparent in plants under stress conditions. In maize crops, application of 0.5mM of salicylic acid improves the growth rate of plant under hydroponic conditions under cold stress (Janda et al., 1997; Janda et al., 1999). It was observed that SA application reduced electrolyte leakage and improves CAT activity with a level of enhancement in the activities of glutathione reductase and guaiacol peroxidase. Application of SA in normal conditions may cause deleterious effects on plants but in stress conditions, it can act positively (Waraich et al., 2011). Likewise in the wheat crop that treatment with salicylic acid at the rate of 0.5 mM can mitigate heat stress damage by increasing the production of proline and reducing the activities of proline oxidase (PROX) which finally leads to maintaining osmotic potential and photosynthetic activities in the plant (Khan et al., 2013). From the result, it was observed that plant tolerance was created SA through interacting with proline activity and ethylene formation and eventually leads to alleviating the photosynthetic damage caused by heat stress in wheat.

## 6 Nutrients and their effect on abiotic stress

Nitrogen is a major component of all cellular and metabolic activities in crop plant as it is a major element of proteins, chlorophyll, nucleic acids, amino acids, plant hormones, enzymes, and osmolytes, all of which are involved in plant abiotic stress tolerance mechanisms through different pathways (Arghavani et al., 2017; Singh et al., 2019). The application of N enhances the plasticity and water extraction capacity of plant roots from the soil, which helps to maintain optimal relative leaf water content and increase water use efficiency in environments with limited moisture (Yang et al., 2012; Tran et al., 2014). Nitrogen supplementation was able in alleviating NaCl-induced toxicity in tomato seedlings which up-regulate the AsA–GSH cycle, K^+^, and K^+^/Na^+^ ratio, which resulted in better growth performance (Nazar et al., 2011). In *Brassicas it was found that* application of N may improves a lot of cellular activities and also prove to be mitigate the ill effects of salt stress in plant. Under the salinity stress condition application of N can improve growth attributes, physio-biochemical parameters, nutritional enrichment, and yield attributes in brassicas (Siddiqui et al., 2010). Application of nitrogen fertilizer to crops promotes antioxidative defense mechanisms and reduces leaf senescence. These processes include carbon partitioning, carbohydrate buildup, cellular membrane stability, and osmoregulation (Saneoka et al., 2004; Saud et al., 2017), cell synthesis and expansion of plant cells (Li et al, 2012), increased photosynthetic capacity (Gessler et al., 2017). N can boost the root system in crops including rice, wheat, rapeseed, and pearl millet as well as improve xylem transport, photosynthetic enzyme activity, antioxidant defense, delay cell senescence, control stomata, increase proline accumulation, and encourage profuse branching (Rostamza et al., 2011; Albert et al., 2012; Tran et al., 2014; Abid et al., 2016.). Under drought conditions, phosphorus promotes root architecture and proliferation in the soil, which increases root volume and hydraulic conductivity (Jin et al., 2015). Application of phosphorous during the early stages of the wheat crop boosted root growth and establishment (Ahmed et al., 2018). The application of P reduces the formation of ROS caused by drought by energizing enzymatic antioxidants as POD, CAT, APX, SOD, and monodehydroascorbate reductase (MDHAR), which consequently increases resistance to stress (Meng et al., 2021). Sardans and Penuelas (2012), P treatment has also been linked to the remodeling of nitrogenous compounds in terms of buildup and absorption of NH_4_ + and NO_3_ in water-stressed agricultural plants. Phosphorus fertilization significantly increased all growth parameters, chlorophyll content, nucleic acid content and minerals content of the common bean plants under salinity stress (Mohamed et al., 2021). Protective effect of potassium application on salt stress in two tomato genotypes (Nasir and Skyland-II) more dry biomass production, shoot K^+^ concentration, chlorophyll contents, stomatal conductance, and K^+^/Na^+^ ratio under saline condition (7.5 dSm^−1^) (Muhammad et al., 2020). Exogenous K fertilizer treatment of 160 kg/ha under water stress enhances grain yield, harvest index, and other physiological indicators in rice (Zain et al., 2014). K can increase the photosynthetic process and glucose metabolism in a stressed cotton crop (Zahoor et al., 2017). In order to reduce abiotic stress in plants, secondary nutrient like calcium is also necessary for food uptake, enzymatic and hormonal up-regulations, and stabilization of cell membranes (Rahman et al., 2015), improves the ability to preserve water (Shao et al., 2008). Ca^2+^ alters the plasma membrane’s level of hydration, which enhances the cohesiveness of the cell walls and raises the viscosity of the protoplasm, enhancing the resistance of cells to dehydration (Ma et al., 2009). Xu et al. (2013) reported that the application of 10 mM Ca in drought conditions caused the production of more root and shoot biomass and dry weight. Magnesium can produce photosynthetic pigments, accumulate higher proline content in mungbean, and encourage better root proliferation in rice (Thalooth and Tawfik Mohamed, 2006; Ding and Xu, 2011). Min et al. (2016) reported that Sulfur helps to nullify the oxidative stress produced due to drought stress by increasing the activities of ROS scavengers like CAT, SOD, and APX; higher H_2_S and soluble sugar contents along with reducing H_2_O_2_. Boron promotes the resistance of crop plants by improving hormone synthesis, lipid metabolism, pollen formation, sugar transport, photosynthetic efficiency, seed germination, flower retention, and seed yield during drought stress (Michael et al., 2016). Under water scarce conditions, B improved water uptake, and nutritional status from the rhizospheric soil by enhancing the growth of more root hairs and mycorrhizae, ROS detoxification process in chloroplasts preventing photooxidative damage hence establishes membrane integrity and improves drought tolerance in plants (Venugopalan et al., 2021). Zn as an important micronutrient has been observed to improve the synthesis of IAA and gibberellic acid (GA3) like plant hormones under moisture stress conditions and thereby improving plumule length and increase shoot dry weight under drought stress. Zn application also helps in a significant expansion in leaf surface area, stomatal conductance, relative leaf water content, and improvement in chlorophyll and accumulation of osmolyte, thus resulting in enhancing cellular growth, plant harvest and prevention the destructive impacts on leaf cell due to moisture deficiencies (Hassan et al., 2020). Spraying with Fe reduces oxidative stress by depleting H_2_O_2_ content along with breakdown of lipid peroxidation activities by accelerating the enzymatic antioxidant mechanisms (CAT, SOD, and GPX) under water scarce situations and also showed a major impact in triggering the quality and resistance of protein under drought stress (Baghizadeh and Shahbazi, 2013; Afshar et al., 2013). While going for role of copper under drought condition, Copper chlorophyllin (Cu-chl) has been proved to be an important modified water-soluble and semi-synthetic bio-stimulant that helps to improve the antioxidative capacity which leads to decreased oxidative stress in plant (Kamat et al., 2000). Cobalt imparts drought tolerance in plants by increasing water use efficiency by reducing the rate of transpiration, further it activates the antioxidant defense mechanisms in plants (Banerjee et al., 2021).

Among the other nutrients, there are several elucidations of the alleviation effects of Si in salt-induced osmotic stress (Zhu et al., 2015) and oxidative stress (Yin et al., 2019). Si-mediated up-regulation of aquaporin gene expression and osmotic adjustment play important roles in alleviating salinity-induced osmotic stress (Zhu et al., 2019). Further foliar application of micronutrients could be useful for improving the nutrient status, root features, and physiological performance of wheat plants (Fouly et al., 2011). Nutrients in combination with phytohormones, it was noted that many plant nutrients can also alleviate water-logging stress and temperature stress. For example, it is reported that application of boron can improves the activity of the antioxidant system significantly and which leads to nullify the toxic effects of ROS produced by heat stress (Waraich et al., 2011). Similarly, selenium (Se) is known for its major role in synthesis of glutathione peroxidase (GPX) and ultimately prevents the plants from the negative impact of ROS (Lobanov et al., 2008). Also, Zn micronutrients can be used to maintain the permeability of cellular membrane and the optimum dose of Zn can mitigate plants from the devastating impacts of heat stress (Peck et al., 2010). **Tables 5**–**7** highlighted the nutrient application in the alleviation of abiotic stresses in plant systems.

**Table 5:** Reports of nutrients involved in mitigating stress in plant.

Types of nutrients	Crop	Impact on plant	References
Nitrogen	Winter rapeseed (*Brassica napus* L.)	Application of nitrogen in winter rapeseed in water logging can avoid the degradation of photosynthetic pigments and ultimately the dry matter accumulation is enhanced	Men et al., 2020
calcium nitrate, potassium nitrate, and tricyclazole	Canola	Application of calcium nitrate, potassium nitrate, and tricyclazole in water logging conditions can enhance the dry weight of plants along with the length of shoots and roots were increased	Habibzadeh et al., 2013
Phosphorus	Wheat	The application of phosphorus in a waterlogged condition is able to increase root establishment and growth under water stress conditions.	Ahmed et al., 2018
Potash	Cotton (*Gossypium hirsutum* L.)	Application of potash in water logging conditions in plants can show improved growth of plants, enhanced photosynthetic pigments, and photosynthetic capacity. It also enhances the uptalking capacity of nutrients in waterlogged plants	Ashraf et al., 2011
	Rice	In water logging, condition higher concentration of K showed improved photosynthetic pigments, non-structural carbohydrates (NSC) contents, and higher activities of antioxidant as well as reduces the activity of lipid peroxidation in waterlogged rice.	Hasanuzzaman et al., 2018
CR urea	Wheat	According to one research in Australia, it was revealed that the application of Controlled Released urea can avoid waterlogging effects of wheat, and grain yield is increased by approximately 20%	Manik et al., 2019
FYM		Application of farmyard manure in waterlogging conditions can enhance grain Fe, Zn, and Cu concentration of paddy which is essential to prevent water stress in plant	Masunaga and Marques Fong, 2018
Boron	Maize	According to research, it was found that Foliar application of boron can able to improve plant growth and mitigate the deleterious effect of maize under waterlogging	Sayed, 1998
Calcium	Rice-Rape rotation field	In Waterlogging condition of rice–rapeseed rotation field production of rapeseed was particularly reduced and it can be mitigated by the application of Calcium peroxide which after reacting with water releases oxygen, which can serve as an excellent supply of oxygen in redox zone.	Wang et al., 2022
Sulphur	Peach	Application of Hydrogen Sulfide (sulfur source) in waterlogging conditions can reduce the damage occured in Peach Seedlings by improving the activities of antioxidation and reducing Ethylene Synthesis	Xiao et al., 2020
Calcium	Pepper	Application of Ca^2+^ in pepper plants improve the photosynthetic capacity, and root growth, and ultimately the biomass is increased in water logging condition along with enhanced antioxidant enzyme and alcohol dehydrogenase activities.	Yang et al., 2016

**Table 6:** Reports of nutrients involved in mitigating cold stress in plant.

Types of nutrients	Crops	Effect	References
Hydrogen Sulfide	Cucumber	Application of sodium hydrosulfide (NaHS, an H_2_S donor) develops cold stress tolerance of cucumber seedlings also the level of auxin is enhanced in the crop.	Zhang et al., 2021
Potash	Carnation	Application of K in high concentrations with irrigation water prevents plant stem damage during low night temperatures in carnation plants.	Kafkafi, 1990
	Potato	In potato plants, during cold stress decreased yield and increased leaf damage were found which can be mitigated by the application of potash in plants.	Grewal and Singh, 1980
	Tomato, Pepper, and Brinjal	Through the application of K, it was observed that total plant yield was increased by 2.4-fold in tomato, 1.9-fold in pepper, and 1.7-fold in brinjal.	Hakerlerler et al., 1997
Phosphorous	Lowland rice	Application of exogenous phosphorus can alleviate low-temperature stress along with p deficiency also it was helpful in shortening day to heading in early and intermediated transplanting crop of rice	Andrianary et al., 2021
Boron	cucumber, cassava, sunflower	Application of boron during cold stress can alleviate the effect of chilling-induced reduction in, membrane fluidity, plasmalemma hydraulic conductivity, root pressure, and water channel activity which leads to a improving in hydrolic conductance of root, uptake of water and nutrient in plant	Huang et al., 2005
Magnesium	Tomato	During low temperatures and high concentrations of K, the risk of Mg deficiency in tomatoes is high. So, the application of magnesium can achieve the normal growth of plants during cold-stress conditions.	Li et al., 2018

**Table 7:** Reports of nutrients involved in mitigating heat stress in plant.

Types of nutrients	Crops	Effect	References
Magnesium	maize and wheat	Application of Magnesium during heat stress of wheat and maize plants can nullify the damage effect by decreasing oxidative cellular damage caused by ROS.	Mengutay et al., 2013
Nitrogen	Spinach	It was observed in spinach both the photosynthetic activity and the light collection ability of the plant is reduced due to low nitrogen content.	Verhoeven et al., 1997.
	Bean	nitrate-grown bean plants had higher tolerance to photodamage than ammonium-grown ones.	Zhu et al., 2000
	Tomato	plant with ammonium application show better tolerance to heat stress than nitrate-applied plants due to the assembly of proline and quaternary ammonium compounds in tomato plant	Rivero et al., 2004
K+Zn+B	Cotton	In cotton increased ability of TNBPP, NSBPP, TSP, RWC, fiber length, fiber strength and fiber fineness were observed due to foliar application of K and Zn followed by B.	Sarwar et al., 2022
Mg	Bean	The antioxidant activities and antioxidant molecules are increased in bean due to the application of Mg	Cakmak and Marschner, 1992
	Maize	The antioxidant activities and antioxidant molecules are increased in maize due to the application of Mg	Tewari et al., 2004
	Pepper	The antioxidant activities and antioxidant molecules are increased in peach due to the application of Mg	Anza et al., 2005
	Mulberry	The antioxidant activities and antioxidant molecules are increased in mulberry due to the application of Mg	Tewari et al., 2006

## 7 Crosstalk with abiotic stress, phytohormones and nutrients

Crosstalk among and between the phytohormones and nutrients has been reported to have important role in abiotic stress alleviation. Auxin being an important phytohormone enhances drought resistance by interacting with other phytohormones. During drought stress, auxin regulates various members of the ACS (1-aminocyclopropane-1-carboxylate synthase) gene family, which is a rate-limiting enzyme in ethylene biosynthesis further increasing resistance against the stress in plants (Colebrook et al., 2014). It was reported that the exogenous application of IAA can enhance ABA and JA content and it can promote the up-regulation of over expression of drought stress-responsive genes (WRKY2, WRKY56, bZIP11, MYB14, DREB2, MYB48, WRKY108715, and RD22), auxin-responsive genes (GH3.9, GH3.1, IAA8) and down-regulation of leaf senescence genes (SAG101 and SAG102) and auxin responding genes (GH3.3, GH3.6, IAA27) which ultimately improves the plant tolerance towards drought stress in white clover (Zhang et al., 2020). Further, during drought ABA accumulation maintains maize primary root elongation by restricting the production of ethylene (Spollen et al., 2000). Furthermore, in drought stress endogenous CK level reduction in the roots also leads to higher concentrations of macro- and micro-elements, such as manganese (Mn), phosphorous (P), or zinc (Zn) (Ramireddy et al., 2018; Nehnevajova et al., 2019). ABA-activated type-A ARR5 magnifies the ABA-mediated response to stress e. Simultaneously, restricts plant growth by repressing CK signaling *via* a negative feedback loop in Arabidopsis (Huang et al., 2018). Further osmotic stress trigger synthesis of CK which down-regulate the genes of ABA synthesis and ABA-mediated responses, which reduces the damage caused by ROS and lipid peroxidation, reduce the senescence ability of leaves and thus improves the abiotic stress tolerance ability of plant and plant growth (Gujjar and Supaibulwatana, 2019). Further, ABI1 and ABI2 which negatively regulate ABA signaling interact with BIN2 and regulate BRs signaling, which ultimately shows stress responses in Arabidopsis (Wang et al., 2018a; Wang et al., 2018b).

The crosstalk is also having an important place in dealing with salinity resistance in plants. The effect of salt stress can be nullified by seed priming with IAA on wheat seed germination and growth *via* regulation the biosynthesis of free salicylic acid induced by auxin and maintaining ionic homeostasis in leaves (Iqbal and Ashraf 2007). Fahad and Bano (2012) observed that during salinity stress plant can produce significant amount of IAA and reduce the synthesis of ABA in maize plants; however, the application of salicylic acid can significantly increase the IAA. Application of auxin restricts the nodes of tiller in rice by biosynthesis of cytokinin in nodes along with down-regulating OsIPT expression (Liu et al., 2011) during salinity stress. CKs play an important role by acting as a bridge in showing the protective role of epibrassinolide and methyl jasmonate in wheat under salinity (Shakirova et al., 2010). Iqbal and Ashraf (2013a) reported a non-consistent effect of GA_3_ priming (150 mg L^-1^) on auxin concentration in wheat genotypes under salinity stress. GA improved the growth of soybean by regulating the level of other phytohormones under salinity (Hamayun et al., 2010), and increased levels of bioactive GA1 and GA4 showed a concurrent decrease in the level of ABA and SA. In brassica, the application of GA in conjunction with nitrogen was helpful in alleviating salinity stress (Siddiqui et al., 2010). Moreover, BRs-mediated stress tolerance in Arabidopsis was linked with ABA, SA, and ETHY pathways (Divi et al., 2010). The BRs act as synergists to GA and IAA during the hypocotyl elongation of Arabidopsis (Tanaka et al., 2003). ABA acts as an antagonist as it repressed the BR-enhanced expression (BEE1, BEE2, and BEE3) proteins (Friedrichsen et al., 2002). Exogenous application of jasmonates (JA) may change the endogenous ABA, which provides a significant hint for understanding the protection mechanisms against salt stress (Kang et al., 2005). Furthermore, foliar application of N fertilizers at the reproductive stage, particularly in leguminous crops, significantly slows the synthesis of abscisic acid with an enhance synthesis of cytokinin production, which promotes cell elongation, nodulation, shoot development, apical dominance, photosynthetic activity, and assimilates translocation to the sink organs under drought conditions (Vries et al., 2016). Likewise, the synergistic regulation of H_2_S with phytohormones such as abscisic acid, ethylene, and salicylic acid can able to regulate the plant stress response (Zhang et al., 2021). It was observed that a balanced application of nutrients can be useful to mitigate cold stress by protecting the cell against freeze-dry death for a limited period of time (Huixia et al., 2018). The plant supplemented with potassium and magnesium provides better protection during a cold injury in the plant. The application of potassium can regulate the closing of stomatal cells, improves water balance, and prevents uncontrolled water loss through the leaves (Danilova et al., 2016m). Also, Magnesium promotes root growth up to a deeper zone of soil and therefore helps ensure that plants can still absorb water from deeper soil layers *via* a well-developed root system, even when the soil is slightly frozen (Danilova et al., 2016m). Whereas, Auxin a plant growth that promotes its synthetic pathway can create thermo-tolerance in crops. During heat and moisture stress conditions, soil cobalt application combined with foliar K and B sprays manifested immense potential to achieve higher black gram production (Banerjee et al., 2021). A similar study was also carried out in *Lathyrus sativus* by the authors that showed the combined application of N, P, and K with Mo improved growth, physiological efficiency, nutrients uptake, and yield ameliorate heat and moisture stress (Banerjee et al., 2021). Combined application of Zn, B, and Si increased plant height, shoot dry weight, number of stems per plant, leaf relative water content, leaf photosynthetic rate, leaf stomatal conductance, chlorophyll content, and tuber yield in potato during salinity stress condition (Mahmoud et al., 2020). Co-application with other plant nutrients like N, P, K, Zn, Si, etc. can be proven beneficial in alleviating salinity, heat, and moisture stress in plants (Akeel et al., 2020). Application of nutrients like K and Ca improves root growth and improve the uptake of water which leads to regulating the stomatal cell and maintaining the plant body temperature during heat stress. The application of micronutrients like B, Mn, and Se can alter the physical, biochemical and metabolic processes in plants in a positive direction to alleviate the adverse effects of heat stress. Combine application of Selenium (Se) and Salicylic acid (SA) can improve tolerance in crops by activating antioxidant production which can eliminate the ROS and make the plant free from membrane damage (Kumari et al., 2022). It was concluded that hormonal balance and their cross-talk with themselves and the nutrients are critical regarding signal perception, transduction, and mediation of stress response in plants.

## 8 Conclusion

In this current review highlighted the comprehensive information on the response of phytohormones, nutrients application and their interaction in crops grown under various abiotic stress conditions. Majority of phytohormones control and sustain the homeostasis inside the cell by detoxifying the ROS and enhancing the antioxidant activities during varied abiotic stress and can enhance tolerance in plants. In drought condition, application of IAA can trigger the activation of other stress-responsive hormones as well as the production of ROS. Enhanced level of ABA in drought condition can alter the guard cell ion transport and stomatal opening which leads to reduced water loss. Cytokinin application increases transcriptional level of *CKX* genes leads to enhanced CKX activity in many plants. Proline activity is enhanced by applying CKs to create salt resistance in plant. It was also concluded that endogenous hormone levels of IAA, zeatin, and GA_3_ is enhanced by application of that exogenous application of BA. In water logging condition, the accumulation of ROS and malondialdehyde levels is reduced by application of CK and GA_3_. The overexpression of ethylene response factor such as GmERF3 of AP2/ERF gene family, leads to improvement in proline content, soluble sugar, and decreases in the accumulation of malondialdehyde to improve drought tolerance in plant. During heat stress, the pollen sterility is the major cause of yield loss, which can be overcome by application of application of ethylene hormone (ethephon) during heat stress conditions. In saline condition by altering the GA levels can enhance seed germination by overproduction of the salt-inducible DDF1 gene (dwarf and delayed flowering 1). It was estimated that application of GA_3_ (200 ppm) in combination with salicylic acid (150ppm) on the soybean plant provides better mitigation effects by improving the number of pod and seed, chlorophyll content in waterlogged conditions. Also, it was observed that methyl jasmonic acid (MeJA) has a positive effect on augmenting tolerance in plants to a high temperature by altering the antioxidant defense mechanism, decreasing chlorophyll loss due to heat, maintaining good water balance in plant and lowering electrolyte leakage in the crop. It was also revealed that application of 0.5mM of salicylic acid improves the growth rate of plant under hydroponic conditions under cold stress condition. Besides, the application of plant nutrients like N, K, Ca, and Mg are also found to reduce the ROS activities through elevating antioxidants quantity that can scavenge the ROS effect and finally leading to the reduction in cell membrane leakage and increase the photosynthetic ability in the plant by recuperating the chlorophyll cells. Hence, it is concluded that the crosstalk with phytohormones and nutrients can complement each other streamlining the antioxidant activities or ROS signaling pathway in cells and improving the tolerance of crop plants. More amalgamated and detailed research is needed with the combined application of hormones and nutrients to precisely understand the mechanism involved.

## Author contributions

RS, writing and conceptualization of the manuscript. SS, drafting of the manuscript, preparing the table and figure. MB, drafting the manuscript, preparing the different tables. GR, editing and critical reviewing of the final version. All authors contributed to the article and approved the submitted drafted version.
